# Suppression of optineurin impairs the progression of hepatocellular carcinoma through regulating mitophagy

**DOI:** 10.1002/cam4.3519

**Published:** 2021-02-18

**Authors:** Shoichi Inokuchi, Tomoharu Yoshizumi, Takeo Toshima, Shinji Itoh, Kyohei Yugawa, Noboru Harada, Hiroyuki Mori, Takasuke Fukuhara, Yoshiharu Matsuura, Masaki Mori

**Affiliations:** ^1^ Department of Surgery and Science Graduate School of Medical Sciences Kyushu University Fukuoka Japan; ^2^ Department of Molecular Virology Research Institute for Microbial Diseases Osaka University Fukuoka Japan

**Keywords:** adaptor protein, autophagy, beta‐oxidation, liver, mitochondria

## Abstract

Autophagy removes damaged organelles to inhibit malignant transformation during tumor initiation. Once a cancer matures, it uses the autophagic pathway as an energy source. Optineurin (OPTN) is an autophagy adaptor protein that recruits microtubule‐associated protein 1 light chain 3, an autophagosome marker, to the autophagosome. Despite studies of the relation between cancer progression and autophagy adaptor proteins, there are no reports to our knowledge of a correlation between hepatocellular carcinoma (HCC) and OPTN. We aimed here to investigate the effects of OPTN expression on HCC progression through autophagy. Immunohistochemistry was used to measure the OPTN expression in the tissues of 141 Japanese patients with HCC. The effects of OPTN expression on HCC progression and mitophagy were assessed using an *OPTN* knockout (KO) cell line in vitro. We used this KO cell line to establish and exploit a mouse model of HCC to determine the effects of OPTN expression on tumor progression. Immunohistochemical analysis showed that patients with elevated expression of OPTN experienced shorter overall survival (OS) and recurrence‐free survival (RFS). OPTN KO cells proliferated relatively slower versus wild‐type (WT) cells in vitro. Western blot analysis showed that mitophagy was suppressed in OPTN KO cells, and ATP synthesis and beta‐oxidation were reduced. The mouse model of HCC showed that OPTN KO cells formed smaller tumors versus WT cells less 10 weeks after implantation. Overall, the present findings suggest that OPTN is a key mediator of mitophagy that contributes to HCC progression through mitochondrial energy production.

## Graphical abstract

Optineurin (OPTN) is required for mitophagic activity as shown by the significant association of OPTN expression levels with mitochondrial beta‐oxidation. Moreover, elevated levels of OPTN were associated with worse prognosis of HCC. Our findings provide a foundation for determining the roles of other autophagy adaptor proteins in the pathogenesis and progression of HCC as well as in other cancers.

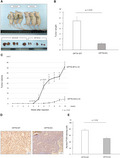

## 1 INTRODUCTION

Hepatocellular carcinoma (HCC) is the fourth and sixth leading causes, respectively, of cancer‐related mortality and morbidity worldwide.^1^ Though surgery remains the most effective therapeutic option for patients with HCC, the majority are diagnosed at an advanced or unresectable stage, or both.^2^ The overall survival (OS) of such patients treated with multi‐kinase inhibitors such as sorafenib, lenvatinib, and regorafenib is approximately 1 year.^3^, ^4^, ^5^ This disappointing outcome may be overcome by increasing our understanding of the complex microenvironment of HCC that contributes to tumor progression.

Autophagy is a catabolic process that degrade the damaged organelles and macromolecules, and is, therefore, called a “self‐eating” process.^6^ Autophagy is initiated by formation of the isolation membrane (double‐membrane phagophore), and the extended phagophore engulfs target organelles and transports them to lysosomes for degradation.^7^, ^8^ After lysosomal degradation, recycling replenishes the cell with nutrients.^9^, ^10^ Our interest is in the association of autophagy with liver diseases.^11^ For example, we found that autophagy is an independent risk factor of recurrence after patients with HCC with large tumors undergo surgery.^12^ Furthermore, hepatocytes use autophagy to provide energy sources that contribute to liver regeneration.^13^ Despite its contribution to the progression of HCC, autophagy is not exploited for clinical intervention.^14^

The selectivity of autophagy for substrates such as mitochondria (mitophagy), viruses (xenophagy), and peroxisomes (pexophagy) is the focus of numerous studies.^15^, ^16^, ^17^ Though microtubule‐associated protein 1 light chain 3 (LC3) is a specific marker of autophagosome formation, proteins accumulated within the autophagosome, which are subjected to selective autophagy, are called autophagy adaptor proteins.^18^ Autophagy adaptor proteins are selectively degraded by autophagy and act as cargo receptors for degradation of ubiquitinated substrates. Certain adaptor proteins have an LC3‐interacting region (LIR) that enables them to polymerize or form aggregates. Furthermore, these adaptor proteins specifically recognize substrates for efficient autophagy.^18^ However, the mechanism underlying the effects of adaptor protein on mitophagy during cancer progression is unknown.

Among these autophagy adaptor proteins, optineurin (OPTN) recruits LC3 to autophagosome in selective autophagic processes such as aggrephagy, mitophagy, and xenophagy.^19^ Mutation of *OPTN* are associated with normal tension glaucoma and amyotrophic lateral sclerosis.^20^ OPTN accelerates the autophagic activities of lung cancer and gastric cancer cells,^21^ and the suppression of OPTN expression reduces the cell division.^22^ In contrast, to the best of our knowledge, the role of OPTN in the progression of HCC through mitophagic activity is unknown. To fill this gap in our knowledge, here we assessed the effects of OPTN expression on the progression of HCC.

## 2 MATERIALS AND METHODS

### 2.1 Patients

Samples from 141 consecutive patients who underwent liver resection for primary HCC without preoperative treatment at our institution between January 2008 and December 2015 were analyzed. The Ethics Committee of our hospital approved this study, in accordance with the ethics guidelines of the Japanese Government (approval number: A30‐281‐0).

### 2.2 Generation of knockout (KO) cell lines

OPTN KO Huh7.5.1 cell lines were generated using CRISPR‐Cas9 technology as previously described.^23^ Briefly, Huh7.5.1 cells were transfected with pX330 encoding hCas9 using *Trans* IT LT‐1 (MIR2306; Mirus) according to the manufacturer's protocol. *OPTN*‐specific single‐guide RNAs (tgAaagctaaataatcaagcc) were inserted into the plasmids at the BbsI site. Single clones were established using a limiting dilution technique. To select KO clones, we determined mutations in the target loci using the Surveyor assay (706020; Integrated DNA Technologies) according to the manufacturer's protocol. Frameshift mutations and inhibition of protein expression were determined using direct sequencing and immunoblotting, respectively.

### 2.3 Immunohistochemistry

Immunohistochemical analysis was performed as previously described.^24^, ^25^ Briefly, samples were fixed in 10% formalin, embedded in paraffin, and cut into 3‐μm‐thick sections. The specimens were deparaffinized in xylene and dehydrated using a graded series of ethanols concentrations. The specimens were then heated (121°C) in Tris‐EDTA buffer (pH9.0) for 15 min (except for PCNA‐stained samples). After antigen retrieval, the specimens were treated with 3% H_2_O_2_ in methanol for 30 min to inhibit the activity of endogenous peroxidase and incubated with 10% goat serum to block nonspecific binding of the immunoreagents. Incubation at 4°C overnight with a primary mouse monoclonal antibody against OPTN (sc‐166576, 1:100; Santa Cruz Biotechnology, Inc.), LC3 (5F10, 1:100; Nanotools Antikorpertechnik), and PCNA (M0879, 1:100; DAKO, Santa Clara, CA, USA) was performed, followed by incubation with the secondary antibody (K4001, EnVision+System‐HRP Labeled Polymer, Anti‐mouse, DAKO). The sections were subjected to the hyper‐sensitive polymer method, and 3,3′‐diaminobenzidine (DAB) was used as the chromogen.

### 2.4 Analysis of immunohistochemical data

The immunoreactivity of OPTN was evaluated according to the German Immunoreactive Score.^26^ Staining intensity was first rated on a scale from 0 to 3, with 0, 1, 2, or 3 indicating no, weak, moderate, or strong, respectively (Figure S1A). Cells (≥500 cells) were counted, and the percentage of positive cells was scored as follows: no staining, 0; 1%–10%, 1; 11%–50%, 2; 51%–80%, 3; and 81%–100%, 4. The final score was calculated by multiplying the staining intensity score derived from the percentage of positive cells to achieve theoretical results from 0 to 12 (Figure S1B). Histological and immunohistochemical evaluations were independently performed by two observers (SI and KY) who were not aware of the clinical characteristics of the patients. To compare the intensity score with OS, the cutoff value was determined using area under the receiver operating characteristic (AUROC) curves. Scores of 0–5 and 6–12 were defined as low and high expression, respectively.

LC3 and proliferating cell nuclear antigen (PCNA) staining were each divided into positive and negative groups. Staining for LC3 was judged positive even if a small area of tissue was stained (Figure S2A). The number and percentage of PCNA‐positive hepatocytes were determined.

### 2.5 Analysis of The Cancer Genome Atlas (TCGA) data

RNA‐seq data of liver hepatocellular carcinoma (LIHC) samples were obtained from the TCGA data portal (https://tcga‐data.nci.nih.gov/tcga/tcga‐Home2.jsp) via bulk download mode [LIHC (cancer type), RNA seqV2 (data type), level 3 (data level), All (preservation), and 1.12.0 (data version)] on October 16, 2016. Sequences were determined using an Illumina Genome Analyzer RNA Sequencing platform. Gene expression data from RNASeqV2 results were quantified using Expectation‐Maximization (RSEM)^27^, ^28^ with the “rsem.gene.normalized_results” file format. Extracted data were applied with no further transformation, expect for rounding values to integers. The cutoff values, which were determined from the AUROC curve, were defined as high (≥5.8 × 10^5^) and low (< 5.8 × 10^5^).

### 2.6 Cell culture

The human HCC cell line Huh7.5.1 (OPTN WT or KO) cells were cultured in Dulbecco's Modified Eagle's Medium (DMEM) (Thermo Fisher Scientific) supplemented with 10% heat‐inactivated fetal bovine serum and 100 mg/ml streptomycin sulfate and incubated at 37°C in an atmosphere containing 5% CO_2_. Cells were starved of amino acids by incubation in D‐MEM (High Glucose) with Sodium Pyruvate, without Amino Acids (Wako Pure Chemical Industries, Ltd.) at the indicated time.^29^

### 2.7 Plasmid transfection and virus infection

To overexpress OPTN, HepG2 cells were transfected with an empty vector (pCMV6‐Entry Vector; Origene) and Myc‐DDK‐tagged‐OPTN (NM_001008211; Origene), respectively, using a JetPrime kit (Polyplus Transfection) following the manufacturer's instructions.^30^ HepG2 were selected with 500 µg/ml G418 (Sigma‐Aldrich) 48 h after transfection. The limiting dilution method was to isolate clones from the transfected HepG2 cells. Single cells were selected and placed in each well of the culture plates, and clonal populations generated from each single cell were isolated. We established three stable clonal lines of OPTN plasmid‐transfected HepG2 cells. All experiments involving virus preparations were performed in accordance with class‐II biosafety procedures. Transduction efficiency was confirmed using western blotting (Figure S7A).

### 2.8 Immunofluorescence analysis

Cells (1 × 10^5^) were cultured on glass slides and fixed with 4% paraformaldehyde in phosphate‐buffered saline (PBS) at room temperature (RT) for 30 min, washed three times with PBS containing 0.2% Triton (35501‐15; Nacalai Tesque), and then, blocked with 10% goat serum for 1 h at RT. The cells were incubated with a primary antibody specific for OPTN (diluted at 1:400) for overnight at 4°C, washed three times with PBS, and then, incubated with a mouse‐Alexa Fluor 488‐conjugated secondary antibody (A10684, Life Technologies) for 1 h, RT. Cell nuclei were stained using Hoechst (Hoechst 33342, Invitrogen). Mitochondria were stained using the Mito‐ID Red Detection Kit (Enzo Life Sciences).^31^ Cells were observed using a fluorescence microscope (Biorevo BZ‐9000, Keyence).

### 2.9 Protein extraction and western blot analysis

Protein extraction and western blot analysis were performed as previously described.^12^ Briefly, iced liver cancer tissues were homogenized using a Beads crusher µT‐12 (Taitec). Homogenized tissues and adherent cells were lysed using RIPA buffer (08714‐04, Nacalai Tesque). Proteins (120 μg) were separated using SDS‐PAGE and then, transferred to polyvinylidene difluoride membranes. The membranes were washed, blocked, and incubated with a primary antibody specific for OPTN (sc‐166576, dilution 1:1000, Santa Cruz Biotechnology, Inc.), LC3 (5F10, 1:100; Nanotools Antikorpertechnik), p62 (ab56416, dilution 1:4000; Abcam), COX‐II (ab110258, dilution 1:1000; Abcam), cyclin D1 (M‐20, dilution 1:400; Santa Cruz Biotechnology, Inc.), and beta‐actin (#4970, dilution 1:2000; Cell Signaling Technology, Inc.), followed by incubation with horseradish peroxidase‐conjugated secondary antibodies (ab6789 or ab6721, dilution 1:500, Abcam). Immunocomplexes were visualized using an enhanced chemiluminescence assay kit (ECL Plus Western Blotting Detection Reagents, GE Healthcare).

### 2.10 Real‐time PCR

Total RNA was extracted from cell lines and frozen tumor specimens using the acid guanidinium thiocyanate/phenol/chloroform method.^12^ After DNase treatment and reverse transcription (RT) using a SuperScript cDNA Synthesis Kit (Invitrogen), real‐time PCR was performed for 35 cycles for 15 s at 95°C and 60 s at 60°C, using a StepOnePlus Real Time PCR System (Applied Biosystems) with a Quantitech SYBR Green PCR kit (Qiagen). Real‐time PCR primers are listed in Table 1. Beta‐Actin was used as an endogenous control to normalize the expression levels. Relative gene expression is expressed as 2^−ΔCt^.^12^

**TABLE 1:** Analysis of the association of OPTN expression with overall survival and recurrence‐free survival of patients with HCC

Variables (*n* = 141)	Overall survival						Recurrence‐free survival					
	Univariate			Multivariate			Univariate			Multivariate		
	HR	95% CI	*p*‐Value	HR	95% CI	*p*‐Value	HR	95% CI	*p*‐Value	HR	95% CI	*p*‐Value
Sex, male	1.38	0.81–2.37	0.23				1.50	1.04–2.17	0.03	1.96	1.06–3.62	0.03
Age ≥60 year	1.63	0.90–2.96	0.11				1.15	0.79–1.67	0.45			
Albumin (g/dl)	0.45	0.30–0.72	<0.01	0.47	0.22–1.06	0.06	0.57	0.42–0.79	<0.01	0.64	0.37–1.08	0.09
Total Bilirubin (mg/dl)	0.52	0.24–1.03	0.06				0.78	0.49–1.21	0.27			
AFP (ng/ml)	1.00	0.99–1.00	0.68				1.00	0.99–1.00	0.82			
DCP (mAU/ml)	1.00	1.00–1.01	0.03				1.00	1.00–1.01	0.01	1.00	1.00–1.00	0.60
ICGR_15_ (%)	1.00	0.99–1.00	0.55				1.00	1.00–1.00	0.19			
Tumor size (cm)	1.07	1.01–1.14	0.03	1.09	0.97–1.22	0.14	1.07	1.02–1.11	<0.01	1.10	1.01–1.19	0.03
Differentiation, poorly	1.78	1.13–2.80	0.02	0.91	0.36–2.29	0.84	1.53	1.11–2.13	0.01	0.57	0.30–1.09	0.09
Microvascular invasion, yes	1.40	0.89–2.20	0.15				1.65	1.22–2.24	<0.01	1.92	1.16–3.20	0.01
Multiple tumors, yes	1.64	1.03–2.62	0.03	2.96	1.30–6.71	0.01	1.95	1.41–2.70	<0.01	2.16	1.25–3.72	<0.01
OPTN, high	3.28	1.55–6.96	<0.01	3.70	1.68–8.15	<0.01	1.79	1.14–2.80	0.01	1.91	1.19–3.08	<0.01

AFP, alpha‐fetoprotein; CI, confidence interval; DCP, des‐γ‐carboxy prothrombin; HCC, hepatocellular carcinoma; HR, hazard ratio; ICGR_15_, 5‐min indocyanine green retention rate; OPTN, optineurin.

### 2.11 Wound healing assay

Cells were grown to 70%–80% confluence. A scratch was introduced into the cell monolayer followed by culture in DMEM. We measured the migration distance 0 h and 12 h after scratching.^32^ Each experiment was independently performed five times.

### 2.12 Transwell migration assay

Huh7.5.1 (OPTN WT and KO) cells (1 × 10^4^, 150 µl) were collected in serum‐free medium and spread onto the upper chamber of a Transwell plate. We filled the lower chamber with 700 µl of medium containing 10% FBS. The plates were incubated at 37°C for 24 h, and the membranes were then fixed in cold methanol and stained with crystal violet.^32^ Each experiment was independently performed six times.

### 2.13 Cell proliferation assay

Cell proliferation was evaluated using a Cell Counting Kit‐8 (CCK‐8; Beyotime Institute of Biotechnology).^32^ The cells were cultivated in 96‐well plates for 0, 1, 2, 3, and 4 days in normal medium. CCK‐8 (10 µl, 5 mg/ml) was added to each well. The cells were incubated for 1 h, and OD_450_ was measured using a microplate reader (Bio‐Rad). OD_450_ values were converted to cell numbers according to a calibration curve.

### 2.14 Colony formation assay

Cells were seeded in 6‐well plates (300 cells per well), cultivated in normal medium at 37°C in an atmosphere containing 5% CO_2_ for 12 days, washed with PBS, and colonies were stained with 0.1% crystal violet for 15 min at RT. The colonies were manually counted, and the images were acquired using a digital camera.^33^

### 2.15 ATP assay

Total cellular ATP concentrations were quantitated using a “Cell” ATP Assay reagent (Wako Pure Chemical Industries, Ltd.). Cells (4 × 10^4^) were added to the wells of 96‐well plates. Six hours before the assay, cells were incubated in starvation medium. Cells were lysed with 100 µl lysis buffer and placed directly into the chamber of a luminometer. Light emission was recorded after addition of 100 µl of luciferin‐luciferase solution.^12^

### 2.16 Cytofluorimetric analysis of mitochondrial transmembrane potential

Cytofluorimetric analysis of mitochondrial transmembrane potential was performed as previously described.^34^ Briefly, changes in mitochondrial transmembrane potential during amino acid starvation were analyzed using 5,5′,6,6′‐tetrachloro‐1,1′,3,3′‐tetraethylbenzimidazolcarbocyanine iodide (JC‐1; Molecular Probes). Cells (1 × 10^6^) were incubated for 30 min in 500 µL of 1 × working solution containing JC‐1. The photomultiplier tube values of FACScan acquisition were 463 V and 642 V for FL1 and FL3, respectively; FL1‐FL2 compensation was 10.8% and 0% for others. Carbonyl cyanide 3‐chlorophenylhydrazone served as the positive control and dimethyl sulfoxide as the negative control (Figure S3A).

### 2.17 Measurement of beta‐hydroxybutyrate

The beta‐hydroxybutyrate concentration of cell lysates were spectrophotometrically determined using a beta‐Hydroxybutyrate (Ketone Body) Assay Kit (Colorimetric) (CELL BIOLABS, Inc.).^35^ Briefly, beta‐hydroxybutyrate dehydrogenase was used to generate a product that reacts with the colorimetric probe that absorbs at 450 nm. The ratio of beta‐hydroxybutyrate to protein weight was calculated according to the manufacturer's instructions.

### 2.18 Measurement of intracellular ROS

Huh7.5.1 (OPTN WT or KO) cells (4 × 10^4^) were added to the wells of a 96‐well plate. The cells were incubated under normal conditions for 48 h and then, in starvation medium for 4 h. ROS were measured using a Cell Meter Fluorimetric Intracellular Total ROS Activity Assay Kit (22901, AAT Bioquest) according to the manufacturer's protocol.^36^ ROS concentrations were determined using a hybrid multimode plate reader Synergy H1 (BioTek) equipped with 485 and 528 nm excitation and emission filters. Cell‐free medium was used to subtract the background.

### 2.19 Mice

Male 5‐week‐old BALB/c‐nu/nu mice, which were purchased from Charles River Inc., were housed in sterilized cages in a vinyl isolator. Mice were provided the CL‐2 diet (CLEA Japan, Inc.) and housed in a pathogen‐free animal facility at 22°C under a 12‐hour light/dark cycle. After 1 week of acclimatization, we subcutaneously injected mice with 3 × 10^6^ each of Huh7.5.1 OPTN WT or KO cells during the light cycle. After 10 weeks, the mice were sacrificed, and the subcutaneous tumors, lungs, and liver were harvested.^33^ These studies were conducted in accordance with the Guide for the Care and Use of Laboratory Animals of the National Institutes of Health and approved by the Animal Committee of our institution (approval number is written in another document).

### 2.20 Statistical analysis

All values are expressed as the mean ± SD. Statistical analyses were performed using the JMP Pro 13 software package (SAS Institute). The significance of the relationship between two variables was determined using Pearson correlation analysis and the Student *t* test. Univariate and multivariate analyses of risk factors of mortality or recurrence of HCC were performed using Cox proportional hazard's model. Survival curves were analyzed using the Kaplan‐Meier method. Generally, ≥3 biological replicates were performed. *p* < 0.05 indicates significant differences.

## 3 RESULTS

### 3.1 High OPTN expression contributes to the progression of HCC

**FIGURE 1:**
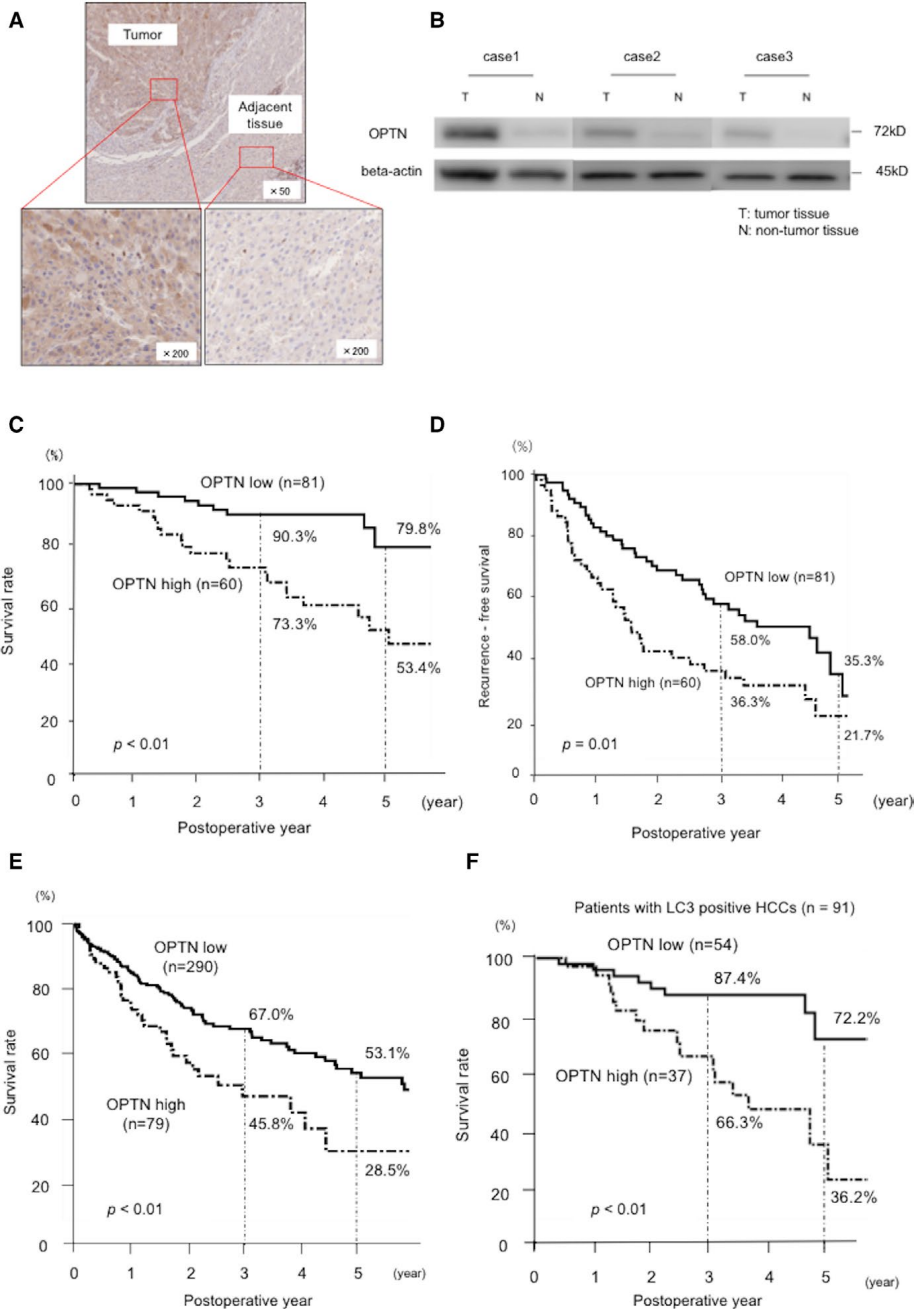
High expression of OPTN contributes to HCC progression. (A) Immunohistochemical analysis of human tissues. Figures show OPTN immunostaining of HCC tissue and adjacent tissues. (B) Western blot analysis of OPTN expression in the three matched HCC tissues (T) and non‐tumor liver tissue (N). Non‐tumor liver tissues were at least 1 cm from tumor tissue. (C and D) Kaplan‐Meier analysis of HCC‐specific survival rates (C) and recurrence‐free survival rates (D) (*n* = 141) between OPTN‐high expression and ‐low expression groups. The OPTN‐high group experienced significantly shorter overall survival (*p* < 0.01) and recurrence‐free survival (*p* = 0.01). (E) Kaplan‐Meier survival curves for all patients with liver hepatocellular carcinomas (LIHCs) (*n* = 369) between OPTN‐high LIHCs and ‐low LIHCs from the TCGA database. (F) Kaplan‐Meier recurrence‐free survival curves for OPTN‐high patients and ‐low patients within LC3‐positive HCCs (*n* = 91). HCC, hepatocellular carcinoma; LC3, microtubule‐associated protein 1 light chain 3; LIHC, liver hepatocellular carcinoma; OPTN, optineurin; TCGA, The Cancer Genome Atlas

Immunohistochemical analysis revealed that OPTN was expressed in the cytoplasm of the cells of 141 resected HCC tissues and at higher levels in cancer versus adjacent tissues (Figure 1A). Western blot analysis revealed as well that tumor tissues expressed higher levels of OPTN compared with those of tissues derived from non‐tumor liver tissues (Figure 1B). OPTN expression was not significantly associated with patients’ clinicopathologic characteristics (Table S1).

Kaplan–Meier analysis indicated that the high OPTN expression was significantly associated with worse OS and RFS compared with the low OPTN expression (OS vs. 3‐year survival = 73.3% vs. 90.3%, respectively; 5‐year survival = 53.4% vs. 79.8%, respectively, *p* < 0.01; RFS vs. 3‐year survival = 36.3% vs. 58.0%, respectively; RFS vs. 5‐year survival = 21.7% vs. 35.3%, *p* = 0.01) (Figure 1C and D). These data are consistent with our analysis of the TCGA database of patients in Western countries with high or low levels of *OPTN* expression as follows: 3‐year survival = 45.8% versus 67.0%, and 5‐year survival = 28.5% versus 53.1%, *p* < 0.01 (Figure 1E).

Cox proportional hazards model analysis revealed that the high expression of OPTN was an independent risk factor of mortality (hazard ratio [HR] =3.70, *p* < 0.01) and recurrence (HR = 1.91, *p* < 0.01) (Table 2). To determine if an autophagic mechanism contributed to the poor prognosis of patients with high levels of OPTN expression, immunohistochemical and Kaplan‐Meier survival analyses were conducted as functions of LC3 expression levels. In patients positive for LC3 expression (*n* = 91), high levels of OPTN experienced significantly shorter OS and RFS compared with those of patients with low levels of OPTN as follows: OS, 3‐year survival = 66.3% versus 87.4%, 5‐year survival = 36.2% versus 72.2%, *p* < 0.01 (Figure 1F); RFS, 3‐year survival = 16.2% versus 50.1%, 5‐year survival = 16.5% versus 36.8%, *p* < 0.01) (Figure S2B). The OS and DFS of LC3‐negative patients with HCC were not significantly different compared with those with high or low levels of OPTN (Figure S2C and D). These results suggest that OPTN contributes to the progression of HCC through an autophagic mechanism.

**TABLE 2:** The association of OPTN expression and clinicopathologic characteristics in HCC patients

Variables	OPTN expression (*n* = 141)		*p*‐Value
	Low (*n* = 81)	High (*n* = 60)	
Gender, male/female	61/20	42/18	0.48
Age (years old)	69 ± 1.3	69 ± 1.5	0.95
HBs Ag positive, *n* (%)	15 (18.5)	12 (20.0)	0.85
HCV Ab positive, *n* (%)	40 (49.3)	29 (48.3)	0.90
AFP (ng/ml)	10,270 ± 6700	10,450 ± 7780	0.99
DCP (mAU/ml)	3760 ± 2670	7100 ± 3160	0.42
AST (U/L)	43 ± 3.5	51 ± 4.0	0.14
ALT (U/L)	42 ± 5.1	48 ± 6.0	0.44
Albumin (g/dl)	3.9 ± 0.1	3.9 ± 0.1	0.99
Total Bilirubin (mg/dl)	0.8 ± 0.03	0.8 ± 0.04	0.76
Prothrombin time (%)	90 ± 1.2	89 ± 1.4	0.42
ICGR_15_ (%)	13 ± 0.8	14 ± 0.9	0.18
tumor size (cm)	4.5 ± 0.4	4.5 ± 0.4	0.99
Differentiation poorly, *n* (%)	16 (19.8)	14 (23.3)	0.66
Microvascular invasion, *n* (%)	35 (43.2)	28 (46.7)	0.68
Multiple tumors, *n* (%)	18 (22.2)	15 (25.0)	0.70
UICC stage ≥3, *n* (%)	39 (48.1)	29 (48.3)	0.98

Abbreviations: AFP, alpha‐fetoprotein; ALT, alanine aminotransferase; AST, aspartate aminotransferase;DCP, des‐γ‐carboxy prothrombin; HBs Ag, hepatitis B surface virus antigen; HCV Ab, hepatitis C virus antibody; ICGR_15_, indocyanine green retention rate at 15 min; OPTN, optineurin.

### 3.2 OPTN KO inhibits the proliferation and migration of HCC cells

**FIGURE 2:**
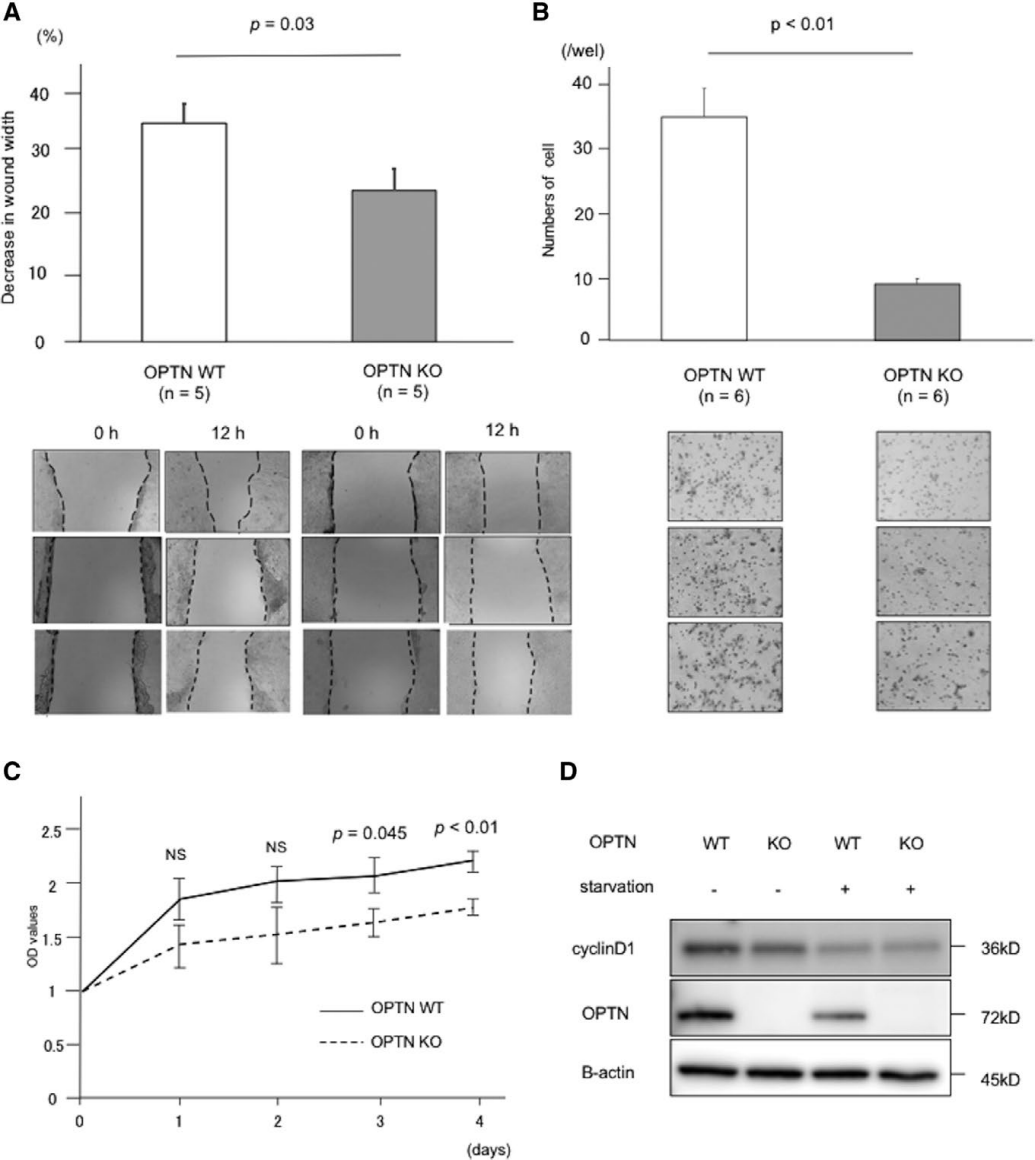
OPTN KO inhibits the proliferation and migration of HCC cells. (A) Representative images of wound healing assays of Huh7.5.1 cell lines. Each experiment was performed for five independent times. (B) Representative images of Transwell migration assays. Each experiment was performed for six independent times. (C) Cell proliferation was evaluated by CCK‐8 assay. After incubation at indicated time, OD_450_ values were measured and converted them to cell numbers. Experiment was performed for six independent times. (D) Western blot analysis of cyclin D1 expression in the KO and WT Huh7.5.1 cell lines. Experiment was performed for three independent times. CCK‐8, cell counting kit‐8

To determine if OPTN contributed to the progression of HCC, we assessed the effects of loss of OPTN expression using the HCC OPTN KO cell line. Wound healing assays revealed that the proliferation and migration of the OPTN KO cells were significantly impaired compared with those of WT cells (*p* = 0.03) (Figure 2A). The Transwell migration assay showed that the ability of OPTN KO cells to migrate was impaired compared with that of the WT cells (*p* < 0.01) (Figure 2B). Moreover, OPTN KO cells formed fewer colonies compared with the WT cells (*p* < 0.01) (Figure S4A and B). The CCK‐8 proliferation assay revealed that the proliferation of OPTN KO cells was significantly impaired 3 and 4 days after incubation commenced (day 3, *p* = 0.045; day 4, *p* < 0.01) (Figure 2C). Western blot analysis revealed that the OPTN KO cells expressed low levels of cyclin D1 compared with the WT cells (Figure 2D).

### 3.3 OPTN KO impairs mitophagy

**FIGURE 3:**
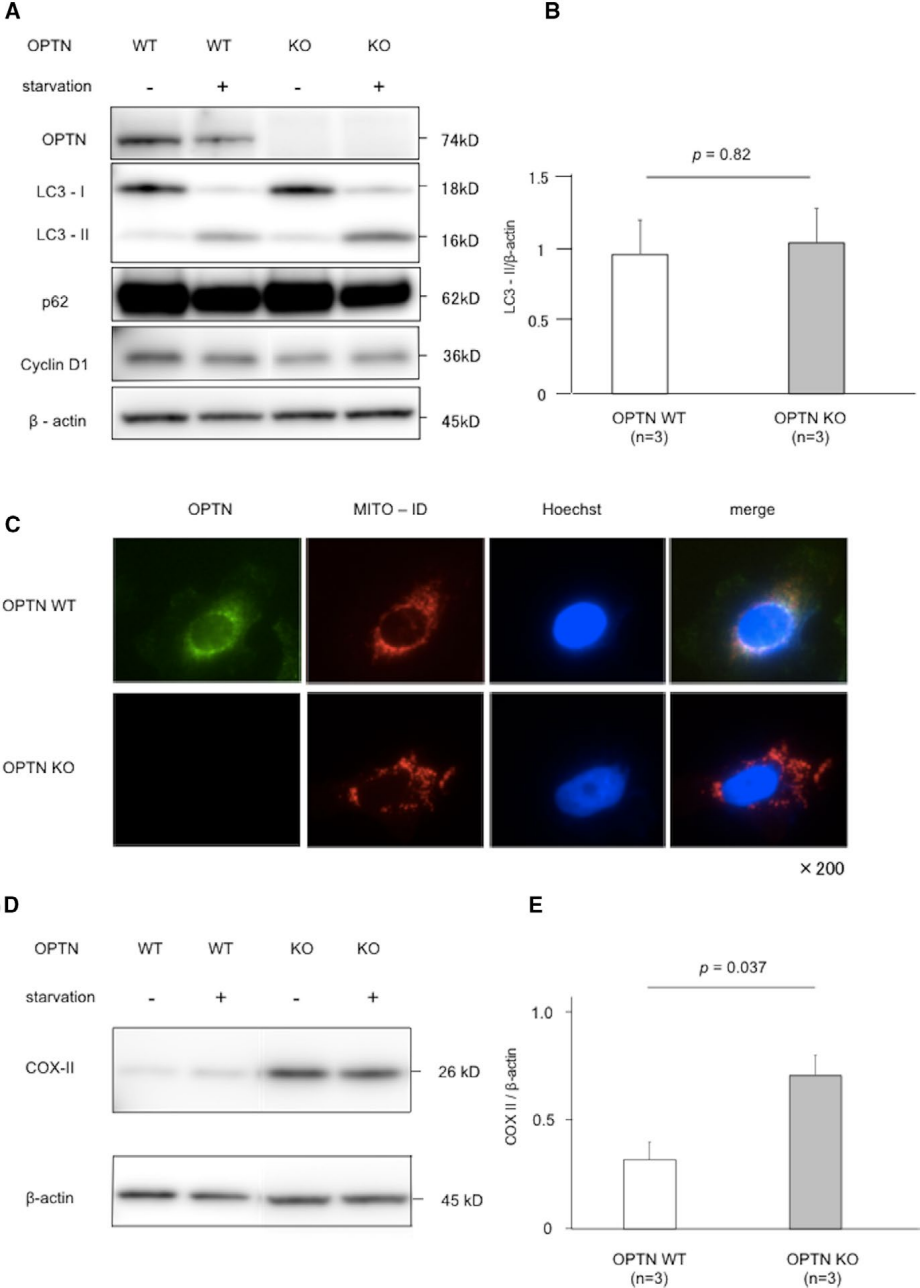
OPTN KO impairs mitophagy. (A and B) Western blot analysis of LC3, p62, cyclin D1 expression in the KO and WT Huh7.5.1 cell lines. Experiment was performed for three independent times. (C) Representative immunofluorescence images of Huh7.5.1 cell line for OPTN and MITO‐ID. Hoechst was used to stain nuclei. (D and E) Western blot analysis of COX‐II expression in KO and WT Huh7.5.1 cell lines. Experiment was performed for three independent times. COX‐II, Cytochrome C oxygenase subunit II; LC3, microtubule‐associated protein 1 light chain 3

It is first hypothesized that the ability of OPTN KO cell to proliferate and migrate was impaired because of the suppression autophagy mediated by OPTN. When Huh7.5.1 cells were starved of amino acids, lipidation of LC3 (conversion of LC3 isotype I to II) increased, and the expression of p62 was decreased in a time‐dependent manner, indicating the induction of autophagy (Figure S5A). Western blot analysis unexpectedly did not detect a significant difference in LC3 lipidation between OPTN WT and KO cells (Figure 3A and B). Furthermore, immunofluorescence analysis localized OPTN to mitochondria (Figure 3C).

Therefore, it is next investigated whether the expression of OPTN correlated with mitophagic activity. Degradation of cytochrome C oxygenase subunit II (COX‐II) is a marker of mitophagy,^37^, ^38^ and we found that this process was impaired in OPTN KO versus WT cells (*p* = 0.037) (Figure 3D and E). These results suggest that OPTN KO cells were deficient in mitophagy, independent of the level of OPTN.

### 3.4 OPTN contributes to ATP production through mitochondrial beta‐oxidation

**FIGURE 4:**
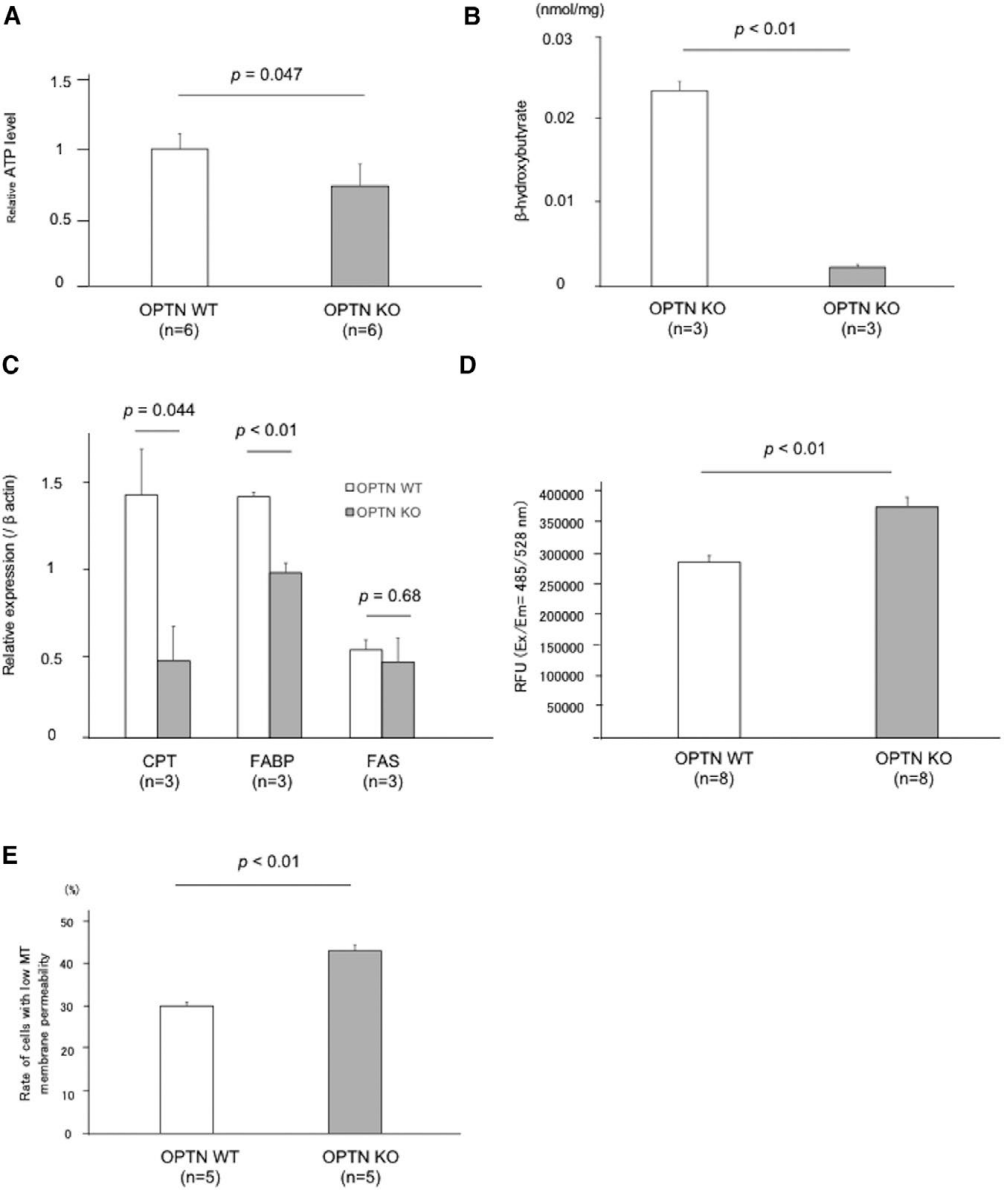
OPTN contributes to ATP production through mitochondrial β‐oxidation. (A) Relative quantification of intracellular ATP levels of OPTN KO and OPTN WT HCC cell lines starved of amino acids cultured under conditions of amino acid starvation. Experiment was performed for six independent times. (B) Quantification of beta‐hydroxybutyrate in the HCC cell lines. Experiment was performed for five independent times. (C) Relative levels of the mRNA encoding CPT, FABP, and FAS. Experiment was performed for three independent times. (D) Quantification of intracellular ROS of OPTN KO and OPTN WT HCC cells lines. Experiment was performed for eight independent times. (E) Rate of HCC cells with low mitochondrial membrane potential in starved condition. Experiment was performed for five independent times. ATP, adenosine triphosphate; CPT, carnitine O‐palmitoyltransferase; FABP, fatty acid‐binding protein; FAS, fatty acid synthase; RFU, relative fluorescence units; MT, mitochondrial membrane potential

OPTN KO cells produced less ATP than WT cells (*p* = 0.047) (Figure 4A). Therefore, it is hypothesized that this result can be attributed to differences in mitochondrial beta‐oxidation. Therefore, the levels of beta‐hydroxybutyrate, the final metabolic product of beta‐oxidation, as well as those of *CPT*, *FABP*, and *FAS*, which are rate‐limiting factors in beta‐oxidation^12^ were measured. The level of beta‐hydroxybutyrate in OPTN KO cells was reduced approximately 10‐fold compared with that of starved OPTN WT cells (*p* < 0.01) (Figure 4B). Similarly, the expression of *CPT* and *FABP* was significantly reduced in OPTN KO cell lines, and *FAS* expression was reduced as well, although the difference as not significant (*CPT*, *p* = 0.044; *FABP*, *p* < 0.01; *FAS*, *p* = 0.68) (Figure 4C).

Furthermore, intracellular ROS levels were significantly higher in OPTN KO cells compared with those of the WT cells when each was starved of amino acids (*p* < 0.01) (Figure 4D). Considering that mitophagy maintains mitochondrial integrity,^29^, ^39^ mitochondrial transmembrane potentials in OPTN WT and KO cell lines were measured. There were more OPTN KO cells with low mitochondrial membrane potential compared with WT cells after amino acid starvation (*p* < 0.01) (Figure 4E, Figure S3A and B). These results suggest that reduced mitochondrial beta‐oxidation in OPTN KO cells impaired clearing of ROS from cells, indicating that OPTN was required mitochondrial integrity.

### 3.5 OPTN KO suppresses tumor progression in a mouse model of HCC

**FIGURE 5:**
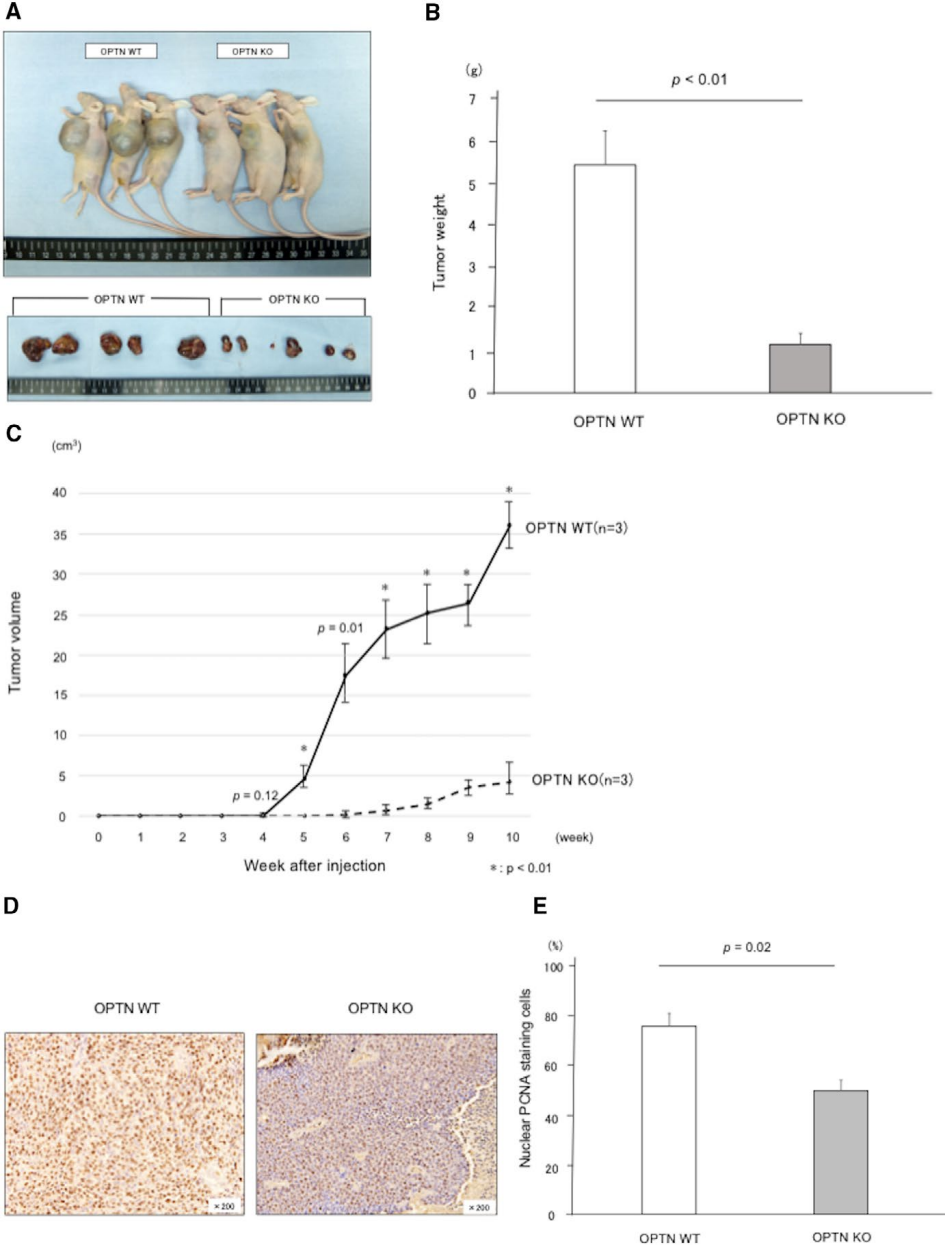
OPTN KO suppresses tumor progression in a mouse model of HCC. (A) Nude mice model of HCC. Mice subcutaneously injected with OPTN WT cells or OPTN KO cells. Subcutaneous tumors were harvested 10 weeks after implantation. Five of the right side were induced by OPTN WT cells and the left six were induced by OPTN KO cells. (B) Compared tumor weight between OPTN WT and KO cells at 10 weeks. (C) Sequential change in volumes of subcutaneous tumors. *p < 0.01. Error bar indicates the SD. (D and E) Typical staining intensities of PCNA in subcutaneous tumors. Error bar indicates the SD. PCNA, proliferating cell nuclear antigen

BALB/c‐nu/nu mice were subcutaneously injected with the OPTN KO and WT HCC cell lines. After 10 weeks, lung and liver metastases were undetectable in either group of mice (Figure S6A and B). Adherent tumors were first observed at the injection site in the mice injected with WT HCC cells after 4 weeks, which promptly enlarged thereafter. Adherent tumors were first observed in the KO mice after 6 weeks, and gradually enlarged thereafter. Tumors formed by the WT cells were significantly larger compared with those formed by the KO cells between weeks 5–10 (*p* ≤ 0.012) (Figure 5A–C). Immunohistochemical analysis of the tumors revealed that tumors formed by the WT cells expressed higher levels of PCNA compared with those formed by the KO cells (*p* = 0.02) (Figure 5D and E), indicating that the WT tumor cells proliferated at a higher rate.

### 3.6 Overexpression of OPTN accelerates the proliferation and migration of HCC cells and increases mitophagy

We next analyzed the proliferation and migration of HepG2 cells engineered to overexpress the OPTN (Figure S7B and C). The migration and proliferation of the OPTN‐overexpressing cells were significantly accelerated compared with the WT‐control cells (*p* < 0.001 and *p* < 0.01, respectively) (Figure S7B and C). Western blot analysis showed that the level of OPTN in the OPTN‐overexpressing cells was higher compared with the control cells (Figure S7A and D). Furthermore, COX‐II degradation increased in the OPTN‐overexpressing cells compared with the control cells.

## 4 DISCUSSIONS

Here it is demonstrated that high OPTN expression was associated with worse prognosis of patients with HCC and that inhibition of OPTN expression suppressed tumor progression through impaired mitophagic activity in a mouse model of HCC and in an HCC cell. Moreover, high OPTN expression was identified as an independent predictor of mortality and tumor recurrence after patients underwent surgery for HCC. OPTN expression levels were significantly associated with mitochondrial beta‐oxidation and mitophagic activity. HCC cells derive energy and nutrients through mitophagy, which likely explains why OPTN promotes the proliferation of HCC cells, leading to poor prognosis of patients after they undergo surgery.

Autophagy suppresses the progression of several cancers.^40^, ^41^ Once a cancer matures, the cancer cells uses autophagy to provide an energy source, and autophagy accelerates tumor progression.^12^, ^14^ Moreover, mitophagy suppresses the activity of the tumor suppressor p53 and facilitates the development of cancer stemness.^40^ In contrast, a little study addresses in detail the relationship between autophagy and p53.^13^ Moreover, we demonstrated that OPTN may play a p53‐independent role in mitophagy and HCC progression.

Deletion of the gene encoding the autophagy adaptor protein p62 from mouse hepatocytes inhibits the proliferation of HCC cells, and that its high expression level in non‐tumor human liver predicts rapid HCC recurrence after curative ablation.^42^ Therefore, we hypothesized that autophagic activity was attenuated in OPTN KO cells. However, to our surprise, the deletion of *OPTN* did not affect the lipidation of LC3, indicating that macroautophagic activity was unaffected. These results are consistent with findings that knockdown of that autophagy adaptor protein nuclear domain 10 protein 52 (NDP52) did not affect the LC3 lipidation compared with the control cells.^43^

These findings may be explained by selective autophagy such as aggrephagy, or peroxophagy that may affect the expression of OPTN. We localized OPTN to the cytoplasm, and OPTN KO cells had reduced mitophagic activity versus OPTN WT cells. COX‐II is a mitochondrial DNA‐encoded inner membrane protein, which is degraded by parkin‐expressing cells subsequent to the induction of autophagy, implicating mitophagic degradation.^37^

We show here that degradation of COX‐II in OPTN KO cells was reduced, indicating impaired mitophagy, which was supported by the level of ROS. Moreover, OPTN KO cells had elevated concentrations of ROS, which supports the conclusion that mitophagic activity was reduced OPTN KO cells, consistent with the scavenging of ROS through mitophagic activity.^44^ For example, OPTN expression increases the COX‐II degradation ratio^37^ and OPTN accumulates ATG13, a component of mitophagy initiating protein, via its LC3‐interacting region and amplifies mitophagy through a positive feedback loop in HeLa cells.^38^ Thus, our results suggest that OPTN contributes to HCC progression through the acceleration of mitophagy that supplies energy through beta‐oxidation.

High expression of p62 shortens the survival of patients with HCC,^42^, ^45^ and evidence indicates that p62 may contributed to the progression of HCC through the mammalian target of rapamycin complex 1 pathway^46^ and the nuclear factor erythroid 2 like 2 (NRF2)‐L‐Kelch Like ECH‐associated protein 1 pathway.^47^ Lower levels of neighbor of BRCA 1 (NBR1) expression is associated with poorer prognosis of clear cell renal cell carcinoma.^48^ Analysis of TCGA data indicates that NBR1 may compete with p62 and that the levels of the mRNA encoding p62 negatively correlate with those of NBR1.^48^

Studies of autophagy adaptor proteins indicate an association between p62 and cancer progression^42^, ^45^, ^46^, ^47^, ^48^, ^49^; however, we are unaware of studies that demonstrate the effect of OPTN on the proliferation of HCC cells. Here we show that high expression of OPTN was associated with worse prognosis of patients with HCC and that the suppression of OPTN expression impaired beta‐oxidation, leading to suppression of the progression of HCC through reduced mitochondrial energy production. Therefore, we conclude that OPTN contributes to cancer progression independent of p62‐mediated pathways.

The autophagy adaptor protein NDP52 is associated with mitophagy.^17^ During mitophagy, NDP52 invades the mitochondrial inner membrane and interacts with mitochondrial RNA poly (A) polymerase (MTPAP) to regulate RNA stability.^50^ Similarly, OPTN is recruited to autophagosomes during mitophagy.^8^, ^19^ Although OPTN accelerates autophagy of cancer cells,^37^ we are unaware of studies into its role in mitophagy. Mitophagy triggers a metabolic switch from oxidative respiration to glycolysis and activates AMP‐activating protein kinase (AMPK) signal during prolonged mitosis.^51^, ^52^ Doménech et al. demonstrated that mitophagy reduced mitochondrial mass and leads to promote AMPK during prolonged mitosis using western blot, flowcytometry, and time‐lapse microscopy.^52^

OPTN catalyzes the autophagic process and regulates the NFκB signaling pathway^53^, ^54^ and prevents polyubiquitination of tumor necrosis factor receptor––associated factor 6 (TRAF6),^54^ which is an adaptor protein of Interleukin‐1 receptor––associated kinase 1 (IRAK1). Polyubiquitination of IRAK1 plays a crucial role during NFκB activation. The AMPK and the NFκB pathways may fill the gap from mitochondria to mitosis and motility. Here we show that OPTN contributed to the progression of HCC, mainly through mitophagy, and that OPTN expression correlated with mitochondrial energy production, particularly beta‐oxidation. These findings suggest that OPTN‐dependent mitophagy activates the AMPK pathway and regulates the NFκB pathway, leading to HCC progression. Identifying the details of the pathway represents a future challenge.

There is a limitation to this study. OPTN is an autophagy adaptor protein that likely interacts with other adaptors such as NDP52‐, NBR1‐, and TAX1‐binding protein 1 during mitophagy. For example, OPTN interacts with p62 to confer resistance to mycobacterial infection of zebrafish.^55^ Further experiments are, therefore, required to identify how these proteins interact.

In summary, we show here that OPTN is required for mitophagic activity as shown by the significant association of OPTN expression levels with mitochondrial beta‐oxidation. Moreover, elevated levels of OPTN were associated with worse prognosis of HCC. Our findings provide a foundation for determining the roles of other autophagy adaptor proteins in the pathogenesis and progression of HCC as well as in other cancers.

## CONFLICT OF INTEREST

The authors declare no conflicts of interest.

## Supporting information

Figures S1–S7‐Table S1

## Data Availability

The data that support the findings of this study are available from the corresponding author upon reasonable request.
