# Supplementary material for: Suppression of optineurin impairs the progression of hepatocellular carcinoma through regulating mitophagy
Source: Cancer Med. 2021 Feb 18;10(5):1501–14. doi: 10.1002/cam4.3519 (PMC7940236; doi:10.1002/cam4.3519)
Supplement: Supplementary file 1 — Figures S1–S7‐Table S1 [file CAM4-10-1501-s001.docx]

**Supporting Information**

Supplementary Figure 1

A: Typical staining intensity of OPTN in HCC. OPTN staining detected in the cytoplasm was classified as strong; +3, moderate; +2, weak; +1, negative; 0 (magnification, ×200). B: Population of each OPTN score.

Supplementary Figure 2

A: Typical staining intensity of LC3. LC3 staining was detected in the cytoplasm, and specimens were classified as LC3-positive or LC3-negative. B: Kaplan-Meier analysis of recurrence-free survival of OPTN-high and -low patients within LC3-positive HCCs. C: Kaplan-Meier analysis of overall survival of OPTN-high and -low patients with LC3-negative HCCs (n = 50). D: Kaplan-Meier analysis of recurrence-free survival of OPTN-high and -low patients with LC3-negative HCCs (n = 50). LC3: microtubule-associated protein 1 light chain 3, OPTN: optineurin

Supplementary figure 3

A: Flow cytometryic analysis of mitochondrial transmembrane potential of Huh7.5.1 cell lines. The horizontal axis represents the number of living cells red fluorescence. B: Flow cytometric images of the mitochondrial transmembrane potential of the OPTN WT and OPTN KO Huh7.5.1 cell lines starved of amino acids. The gated upper and lower sections show the percentages of living and apoptotic cells, respectively. CCCP: carbonyl cyanide 3 - chlorophenylhydrazone, DMSO: dimethyl sulfoxide.

Supplementary figure 4

A, B: Clonogenic assay of Huh7.5.1 OPTN WT or OPTN KO cells. Experiment was performed for five independent times.

Supplementary figure 5

A: Western blot analysis of LC3 and p62 expression in Huh7.5.1 OPTN WT and OPTN KO cell lines starved of amino acids after 0, 1, 2, 3, 4, and 6 h. Experiment was performed for three independent times.

Supplement figure 6

A, B: Appearance and HE staining of lungs and liver of mice inoculated with OPTN WT or OPTN KO cells. Metastasis were not detected.

Supplement figure 7

OPTN overexpression promotes the proliferation and migration of HCC cells.

A: An OPTN-overexpressing HCC cell line (HepG2) was well established.

B: The result of Transwell migration assays. Each experiment was performed for three independent times. C: Cell proliferation was evaluated by CCK-8 assay. After incubation at indicated time, OD_450_ values were measured and converted them to cell numbers. Experiment was performed for five independent times. D: Western blot analysis of COX-II expression in overexpression and WT HepG2 cell lines. CCK-8: cell counting kit-8, COX-II: Cytochrome C oxygenase subunit II.

**Supporting information**

Fig. S1


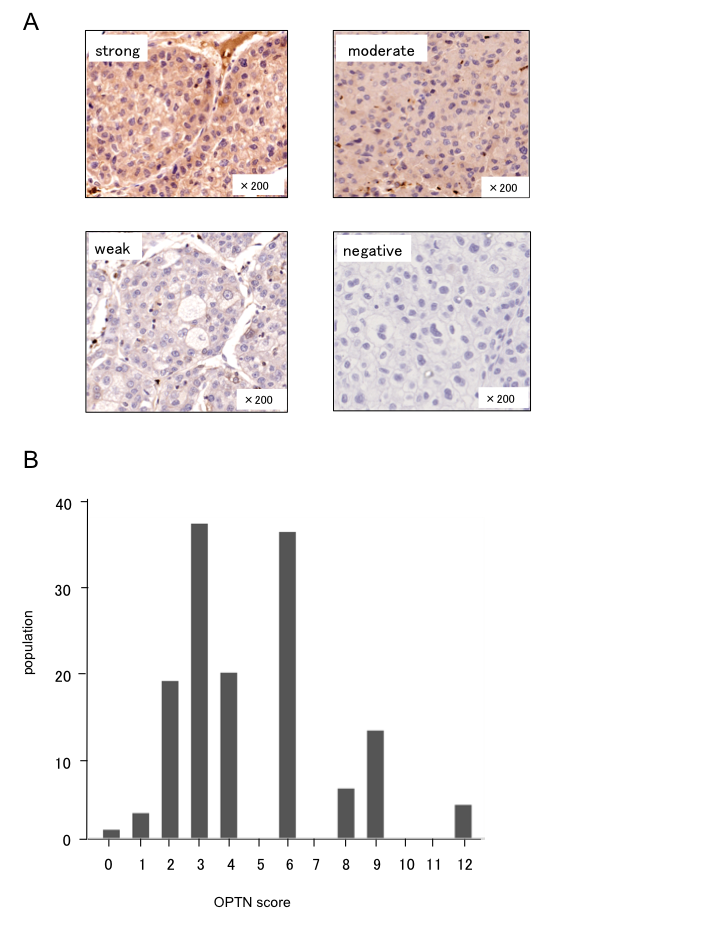


Fig. S2


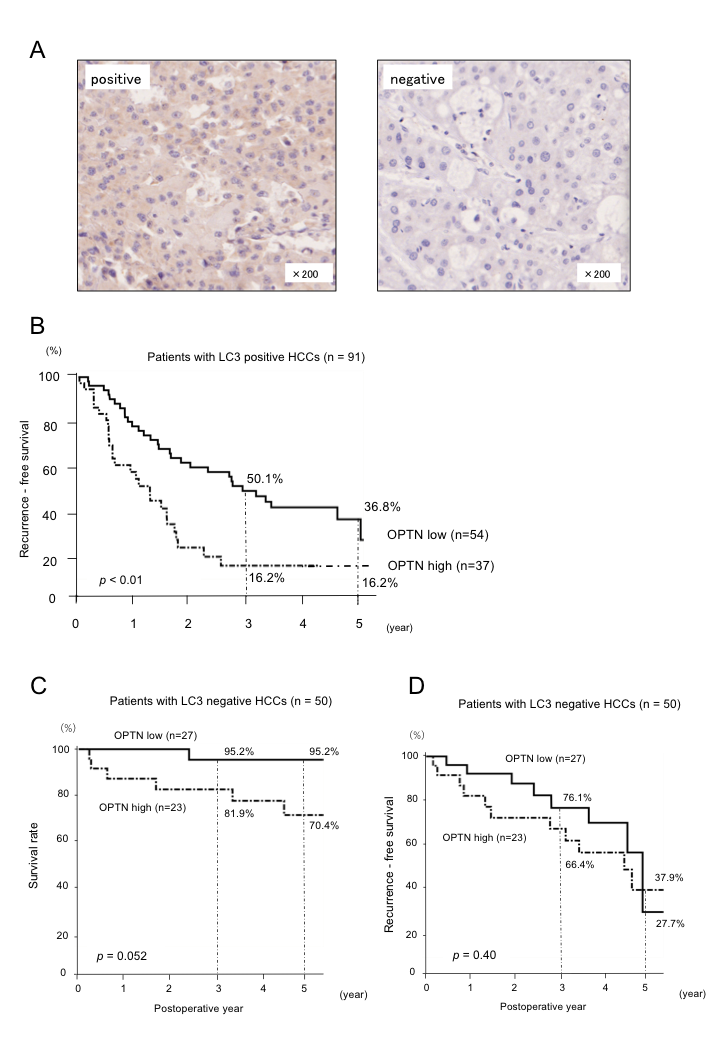


Fig. S3


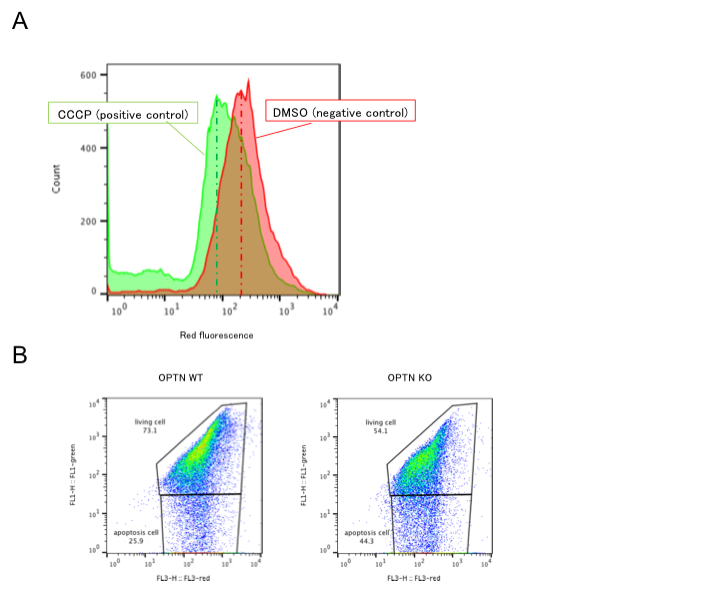


Fig. S4
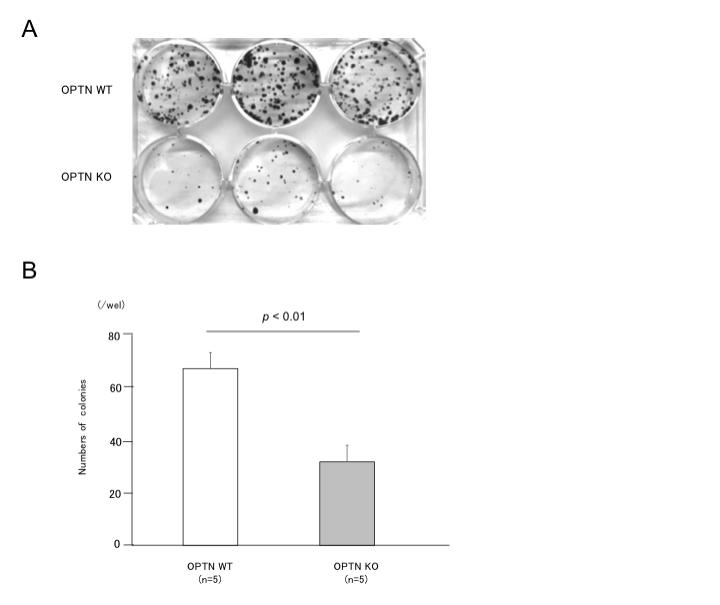


Fig. S5
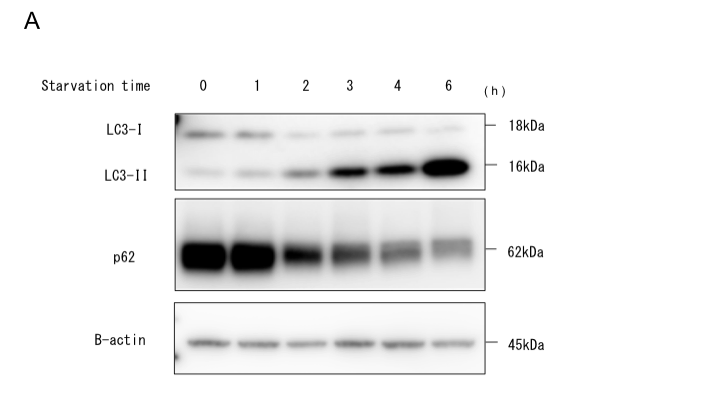


Fig. S6


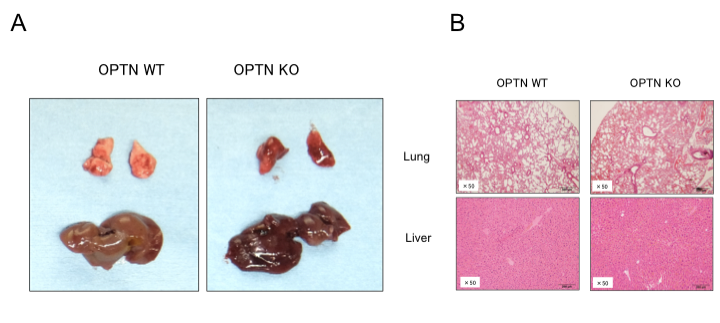


Fig. S7


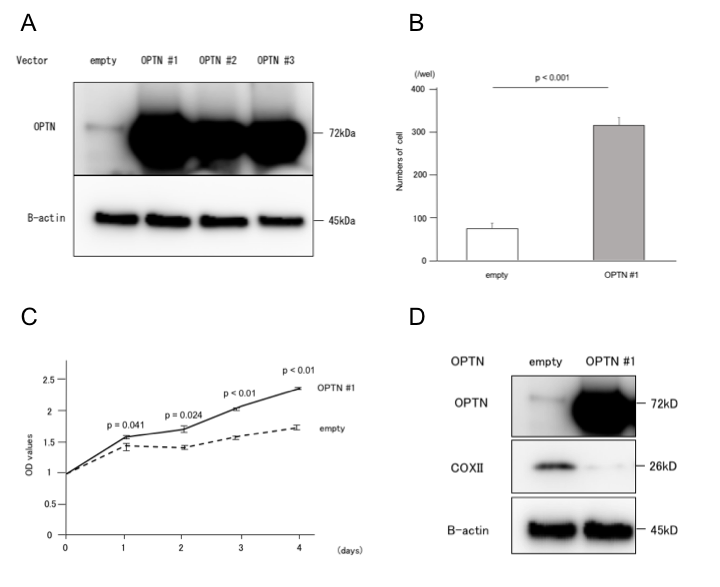


Table S1: The association of OPTN expression and clinicopathologic characteristics in HCC patients.

| variables | OPTN expression (n = 141) | | p value |
| --- | --- | --- | --- |
|  | Low (n = 81) | High (n = 60) |  |
| Gender, male / female | 61 / 20 | 42 / 18 | 0.48 |
| Age (years old) | 69 ± 1.3 | 69 ± 1.5 | 0.95 |
| HBs Ag positive, n (%) | 15 (18.5) | 12 (20.0) | 0.85 |
| HCV Ab positive, n (%) | 40 (49.3) | 29 (48.3) | 0.90 |
| AFP (ng/ml) | 10,270 ± 6,700 | 10,450 ± 7,780 | 0.99 |
| DCP (mAU/ml) | 3,760 ± 2,670 | 7,100 ± 3,160 | 0.42 |
| AST (U/l) | 43 ± 3.5 | 51 ± 4.0 | 0.14 |
| ALT (U/l) | 42 ± 5.1 | 48 ± 6.0 | 0.44 |
| Albumin (g/dl) | 3.9 ± 0.1 | 3.9 ± 0.1 | 0.99 |
| Total Bilirubin (mg/dl) | 0.8 ± 0.03 | 0.8 ± 0.04 | 0.76 |
| Prothrombin Time (%) | 90 ± 1.2 | 89 ± 1.4 | 0.42 |
| ICGR_15_ (%) | 13 ± 0.8 | 14 ± 0.9 | 0.18 |
| tumor size (cm) | 4.5 ± 0.4 | 4.5 ± 0.4 | 0.99 |
| Differentiation poorly, n (%) | 16 (19.8) | 14 (23.3) | 0.66 |
| Microvascular invasion, n (%) | 35 (43.2) | 28 (46.7) | 0.68 |
| multiple tumors, n (%) | 18 (22.2) | 15 (25.0) | 0.70 |
| UICC stage ≥ 3, n (%) | 39 (48.1) | 29 (48.3) | 0.98 |

AFP: alpha - fetoprotein, ALT: alanine aminotransferase, AST: aspartate aminotransferase, DCP: des - γ - carboxy prothrombin, HBs Ag: hepatitis B surface virus antigen, HCV Ab: hepatitis C virus antibody, ICGR_15_: indocyanine green retention rate at 15 min, OPTN: optineurin.
